# Pain Intensity and Sensory Perception of Tender Points in Female Patients with Fibromyalgia: A Pilot Study

**DOI:** 10.3390/ijerph18041461

**Published:** 2021-02-04

**Authors:** Edurne Úbeda-D’Ocasar, Juan Antonio Valera-Calero, Juan Pablo Hervás-Pérez, Mario Caballero-Corella, Cristina Ojedo-Martín, Gracia María Gallego-Sendarrubias

**Affiliations:** 1 Department of Physical Therapy, Universidad Camilo José Cela, Villanueva de la Cañada, 28692 Madrid, Spain; eubeda@ucjc.edu (E.Ú.-D.); jphervas@ucjc.edu (J.P.H.-P.); mcaballero@ucjc.edu (M.C.-C.); cojedo@ucjc.edu (C.O.-M.); gmgallego@ucjc.edu (G.M.G.-S.); 2 Escuela Internacional de Doctorado, Universidad Rey Juan Carlos, 28933 Alcorcón, Spain

**Keywords:** fibromyalgia, pain perception, pain threshold, physical examination, pain

## Abstract

Fibromyalgia syndrome (FMS) is a condition that courses with chronic pain, fatigue, sleep disturbance, impaired quality of life and daily function. Due to the lack of blood, imaging or histological confirmatory tests, the diagnosis of FMS is based on the presence of widespread pain and presence of tender points (TPs). Our aim was to assess the pain pressure thresholds (PPTs) and subjective pain perception (SPP) of all 18 TPs while applying a normalized pressure in female patients with fibromyalgia. An exploratory descriptive pilot study was conducted in 30 female patients with FMS. Sociodemographic data (e.g., age, height, weight, and body mass index), clinical characteristics (e.g., years with diagnosis and severity of FMS), PPTs (assessed with an algometer), and SPP (assessed with a visual analogue scale) of all 18 TPs were collected. A comparative analysis side-to-side (same TP, left and right sides) and between TPs was conducted. No side-to-side differences were found (*p* < 0.05). Significant differences between all 18 TPs were found for PPTs (*p* < 0.0001), and SPP (*p* < 0.005) scores were found. The most mechanosensitive points were located in the second costochondral junction, the occiput, the trochanteric prominence; the most painful while applying a normalized pressure considering the TP and side were those located in the gluteus, trochanteric prominence, and supraspinatus. The current study describes PPTs and SPP, as assessed with algometry and visual analogue scale, respectively, of all 18 TPs in female patients with FMS. TPs exhibited significant PPTs and SPP differences between TP locations with no side-to-side differences.

## 1. Introduction

Fibromyalgia syndrome (FMS) is a condition that courses with generalized chronic pain [1] associated with fatigue, sleep disturbance, depression and cognitive impairments [2,3,4]. FMS affects 2.3–2.4% of the Spanish population and up to 6.6% of the entire world, being more prevalent in rural environments than urban areas [5]. This disease can affect people at different ages, but FMS is most frequently found in middle-aged women [6]. In addition to gender and age, prior family history of FMS increases the risk of suffering this condition. Therefore, this suggests a mixed genetic and lifestyle etiology [7], but the exact etiology is still unknown. Previous studies assessed the altered pain prosecution, reporting a chronic and increased pain response to a painful stimulus (hyperalgesia) and pain caused by a stimulus that normally should not cause pain (allodynia) [8,9,10]. Gender differences were found in the intensity, frequency, duration, and locations, with females more affected than male patients [11]. Although pain perception is conditioned by subjective and personal experiences, these differences between genders could be explained by biologic features related to endogenous pain-relief mechanisms or the influence of gonadal hormones [12].

Due to the lack of confirmatory diagnosis by using imaging, blood, or histological test, FMS is diagnosed on the basis of the presence of a pain response when applying approximately 4 kg/cm^2^ in at least 11 out of 18 tender points (TPs) in musculoskeletal structures and chronic widespread pain with 88.4% sensitivity and 81.1% specificity [13]. These criteria were updated and defined by the American College of Rheumatology (ACR) in 2010 [14], including the presence of widespread pain and substantial somatic symptoms for at least 3 months and exclusion of any other disorder explaining the pain. Widespread pain is defined as the presence of (1) pain on both sides of the body, (2) pain above and (3) below the waist and axial skeletal pain, and is directly related with the number of tender points [15].

However, there is no evidence of normative pain-pressure threshold scores of TPs in patients with FMS, differences between points, and subjective pain perception after applying a normalized pressure. Therefore, the aims of this study were to assess the pain pressure thresholds of all 18 TPs and to evaluate the subjective pain perception applying a normalized pressure in each point in female patients with fibromyalgia.

We hypothesized that we would find both significant pain-pressure thresholds and subjective pain perception after applying normalized pressure differences among all 18 TPs since pain is a complex and multifactorial experience.

## 2. Materials and Methods

### 2.1. Participants

A consecutive sample of female volunteer patients with fibromyalgia who responded to local announcements from October–December 2019 in the Association of Fibromyalgia “AFINSYFACRO” located in Móstoles (Spain) were invited to participate in this exploratory descriptive pilot study. To be eligible to participate, individuals had to be 35 to 70 years old, and have a diagnosis of fibromyalgia at least 3 months prior to their participation in the study. Exclusion criteria included any recent surgery, skin conditions, neuropathic conditions, being under pharmacological treatment (e.g., analgesics or muscle relaxants) three days before their participation, or any underlying medical condition (e.g., tumor or fracture). All participants signed a written informed consent prior to their participation. This study was approved by the Institutional Review Ethical Committee of Camilo José Cela University of Madrid (Spain) with identification number (CEI-UCJC-25/06/2019) and followed the Strengthening the Reporting of Observational Studies in Epidemiology (STROBE) guidelines and checklist [16].

### 2.2. Procedures

The data collection was planned before the physical exam. Before the start of the evaluation, an assessor with more that 10 years of experience treating patients with FMS underwent training to standardize the examination techniques and the interpretation of algometry. This assessor performed a standardized history with sociodemographic data (e.g., age, height, weight, and body mass index (BMI = kg/m^2^)) and clinical information (e.g., pain duration and fibromyalgia severity) and a physical examination including pain pressure thresholds (PPTs) and subjective pain perception (SPP) of all 18 TPs.

Severity of FMS was assessed by using the Spanish version of the fibromyalgia impact questionnaire (FIQ). This questionnaire showed an acceptable reliability (ICC = 0.81) and consists of 10 subscales assessing physical function, number of days feeling good, work missed, ability to do work, pain, fatigue, rest, stiffness, anxiety, and depression. The score ranges from 0 to 100, where a greater score involves more disability and severity [17].

**Figure 1:**
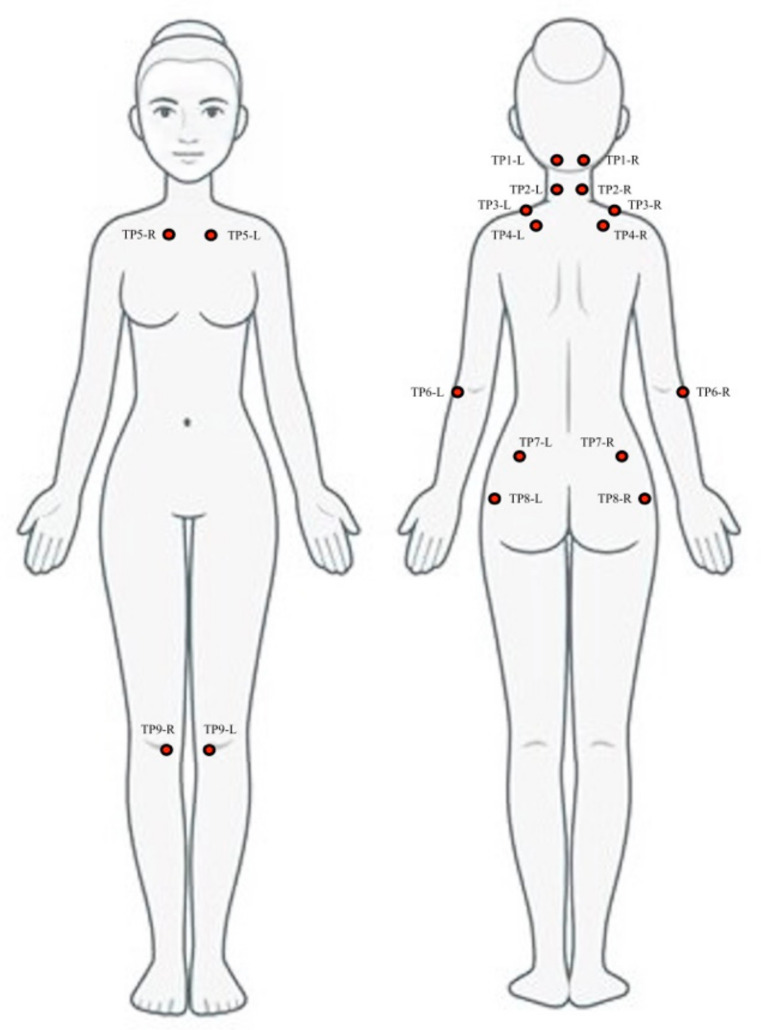
Examined locations of Tender Points assessed in this study. L: Left; R: Right.

The assessment of PPTs in TPs showed a moderate to high intra-examiner and inter-examiner reliability (Cronbach’s α= 0.72–0.76 and 0.70–0.71, respectively) [18] and is considered a valid tool for assessment of fibromyalgia severity [19]. All 18 TPs were assessed by using an analogic algometer Fischer FPN100. Nine locations were bilaterally examined by the same experienced assessor as follows: (1) TP1 located in the forehead, just below the hair line; (2) TP2 located in the anterior aspects of intertransverse space at C5–C7 level; (3) TP3 located in the midpoint of upper fold of trapezius muscle: (4) TP4 located in the supraspinatus muscle above the medial border of the spine; (5) TP5 located just lateral to the second costochondral junction; (6) TP6 located 2 cm distal to lateral epicondyle; (7) TP7 located in the upper outer quadrants of buttocks; (8) TP8 located just posterior to the trochanteric prominence; and (9) TP9 located in the medial fat part of the knee (Figure 1).

All the procedures were performed by the same assessor calculating the mean of two procedures. A maximum of 4 kg/cm^2^ of pressure was applied, increasing at a rate of 1 kg/second. Standardized instructions given to each subject were, “I am going to push on your body at 9 places. If you feel pain, not pressure, say ‘now’ and I will stop”. The assessor recorded algometer readings for PPT at each tender point site on a body chart and calculated the total tender point count.

Finally, for assessing the SPP, patients were asked to identify their level of pain in a 100 mm visual analogue scale (VAS), where 0 was absence of pain and 100 was the worst imaginable pain [20] while applying the correspondent normalized pressure for each side with the algometer, to identify the two most painful points. This normalized pressure was calculated as the mean PPTs for each TP.

### 2.3. Sample Size Calculation

Based on a previous study conducted by Ge et al. assessing the predetermined sites of examination for the same 18 TPs assessed in this study [21], a minimum sample size of at least 30 patients with FMS could be considered as appropriate for this pilot study.

### 2.4. Statistical Analysis

Data were analyzed using SPSS for Windows (v.22, IBM, Armonk, NY, USA). Data distribution was assessed by using the Shapiro–Wilk test. Descriptive statistics (mean and standard deviation for normal variables and median and interquartile ranges for non-normal variables) were calculated for sociodemographic, clinical, PPTs, and SPP characteristics. A t-test for independent samples was conducted to assess side-to-side differences for PPTs and SPP. Finally, a multiple comparison analysis between TPs for PPTs and SPP was conducted using the analysis of variance (ANOVA) test.

## 3. Results

From a total of 33 female patients with FMS, 3 were excluded due to the use of analgesics or muscle relaxants the day of the data collection. Thus, 30 women with FMS were finally included in this study. Table 1 provides sociodemographic and clinical features. The included sample showed a normal distribution for all sociodemographic features.

Table 2 summarizes the data obtained during the physical examination of all TPs, both PPTs and SPP. No significant side-to-side differences were found for either PPTs or SPP (*p* > 0.05). However, significant differences between TPs were found for both PPTs (F = 8.392; *p* < 0.0001) and SPP (F = 2.920; *p* < 0.005). Although the most painful points were located at TP7 (69.6 ± 19.4), TP8 (68.0 ± 21.5), and TP4 (65.1 ± 21.1), the lowest PPTs scores were found at TP5 (1.28 ± 0.42), TP1 (1.52 ± 0.34), and TP8 (1.61 ± 0.59).

## 4. Discussion

This is, to our knowledge, among the first investigations assessing the subjective pain perception of TP while applying a normalized pressure in female patients with FMS. Our data suggest significant PPTs and SPP score differences among the 18 TPs assessed, but we found no between-sides PPTs nor SPP differences for the same TP location. In a decreasing mechanosensitive order, the points were those located in the second costochondral junction (TP5), the occiput (TP1), the trochanteric prominence (TP8), the lower cervical spine (TP2), the knee (TP9), the epicondyle (TP6), the upper trapezius (TP3), the gluteus (TP7), and supraspinatus (TP4), respectively. The most painful perceptions applying a normalized pressure were located in the gluteus (TP7), trochanteric prominence (TP8), supraspinatus (TP4), upper trapezius (TP3), second costochondral junction (TP5), epicondyle (TP6), occiput (TP1), lower cervical spine (TP2), and knee (TP9), respectively, in decreasing order.

A correct clinical assessment during FMS diagnosis is essential, and it should include a comprehensive history including self-reported questionnaires related to the FMS impact (Fibromyalgia Impact Questionnaire), functional capacity (Fibromyalgia Health Assessment Questionnaire), life quality (e.g., SF-12, SF-36, or EuroQol-5D), pain characteristics (S-LANSS, painDETECT, Central Sensitization Inventory), and psychological features (Hospital Anxiety and Depression Scale, Brief Hypervigilance Scale, Pain Catastrophizing Scale, Tampa Scale for Kinesiophobia, Pittsburgh Sleep Quality Index) [2]. However, a proper physical examination is also required for classifying chronic pain conditions, understanding the longitudinal course and severity of the pain disorder [20]. Although timing, location, and distribution of pain are important during the physical examination, sensory and affective qualities of pain should be considered since pain is a complex, internal, and private experience [22].

Our study provides preliminary data of PPT in all 18 TPs in a cohort of female patients with FMS with similar sociodemographic and clinical characteristics. TPs manual palpation and identification can be used to discriminate patients with FMS from healthy subjects [23]. A prior study investigating PPT scores [18] reported that the PPT of TPs in patients with FMS are heterogeneous and reflect individual differences in mean tenderness thresholds and individual profiles of TPs. Thus, Tastekin et al. [23] also reported discriminative values of all tender points. Our results are consistent with these studies since we found PPT significant differences between TPs, but no differences between right and left sides. Based on these findings, the application of a constant pressure of 4 kg/cm^2^ to confirm the presence of a TP should be revised, since the PPTs are different for each point and that pressure is more than two times the pressure necessary to induce pain.

We also find that the most mechanosensitive points were not the most painful when applying a normalized pressure for each point and side. One likely reason explaining this finding could be the complexity of the experience of pain. Several studies are consistent with the role of psychosocial and biological factors in the development of persistent pain and disability [24]. Demographic factors do not directly influence pain perception but represent valuable individual difference factors [25]. Several examples of epidemiologic evidence demonstrated that chronic pain conditions (e.g., migraine, tension-type headaches, fibromyalgia, temporomandibular disorders or widespread pain) are more prevalent among women than men [26]. Thus, many of the biological changes that underlie aging (e.g., systemic inflammation, oxidative stress, altered autonomic function, and changes in neuronal structure and function) can also contribute to increased clinical pain [27]. Therefore, due to the complexity of the biopsychosocial background that influences pain, a multidisciplinary management including educational interventions to enhance provider understanding of individual differences in pain could enhance pain care.

Finally, we have to recognize some limitations of the current study. First, only female individuals were included to determine the PPTs and the SPP, and our results cannot be applicable to male patients with FMS. Second, our sample size was small, and therefore our results should be considered preliminary normative data of PPTs and SPP; further research with a larger sample size is needed to confirm these findings. Finally, we did not include a control group to compare PPTs and SPP scores. Further research is needed to confirm these pain domains differences for diagnosis purposes.

## 5. Conclusions

This study describes PPTs and SPP of all 18 TPs in female patients with FMS. TPs showed differences for PPTs and SPP. Our results suggested that the most mechanosensitive TPs were those located in the second costochondral junction (TP5), the occiput (TP1), and the trochanteric prominence (TP8); the most painful while applying a normalized pressure considering the TP and side were those located in the gluteus (TP7), trochanteric prominence (TP8), and supraspinatus (TP4). Thus, no side-to-side differences were found. However, further research with a larger sample size is needed to confirm these findings.

## Figures and Tables

**Table 1:** Sociodemographic and clinical data of the sample.

	Total Sample (n = 30 Women)
Sociodemographic data	
Age (years)	55.1 ± 8.7
Weight (kg)	68.4 ± 12.61
Height (m)	1.60 ± 0.07
BMI (kg/cm^2^)	26.58 ± 4.36
Clinical data	
Years with diagnosis of FMS	12.1 ± 9.85
FIQ (0–100)	64.1 ± 14.4

Data scores are expressed as mean ± standard deviation.

**Table 2:** Physical examination of Tender Points: Pain Pressure Thresholds and Subjective Pain Perception.

	TP1	TP2	TP3	TP4	TP5	TP6	TP7	TP8	TP9
**Mean (n = 30; 120 measurements)**									
PPT (kg/cm^2^) *	1.52 ± 0.34	1.64 ± 0.45	1.78 ± 0.45	1.90 ± 0.37	1.28 ± 0.42	1.75 ± 1.53	1.87 ± 0.52	1.61 ± 0.59	1.69 ± 0.49
SPP (0–100) ^†^	59.0 ± 24.4	57.8 ± 24.6	62.1 ± 18.9	65.1 ± 21.1	59.3 ± 23.7	59.3 ± 26.3	69.6 ± 19.4	68.0 ± 21.5	54.5 ± 22.8
**Left side (n = 30; 60 measurements)**									
PPT (kg/cm^2^)	1.54 ± 0.38	1.60 ± 0.47	1.76 ± 0.54	1.87 ± 0.37	1.25 ± 0.46	1.76 ± 0.52	1.80 ± 0.54	1.57 ± 0.58	1.73 ± 0.37
SPP (0–100)	59.0 ± 23.3	58.0 ± 23.4	63.3 ± 20.5	65.3 ± 19.7	58.6 ± 22.3	63.0 ± 23.8	68.0 ± 21.2	66.6 ± 22.7	56.0 ± 20.2
**Right side (n = 30; 60 measurements)**									
PPT (kg/cm^2^)	1.5 ± 0.35	1.69 ± 0.47	1.80 ± 0.44	1.92 ± 0.43	1.31 ± 0.47	1.75 ± 0.66	1.93 ± 0.60	1.65 ± 0.70	1.65 ± 0.59
SPP (0–100)	59.0 ± 25.7	57.6 ± 26.2	61.0 ± 17.4	65.0 ± 22.8	60.0 ± 25.4	55.6 ± 28.6	71.3 ± 17.7	69.3 ± 20.4	53.0 ± 25.4

Data scores are expressed as mean ± standard deviation. * Significant Differences between Tender Points (*p* < 0.0001). ^†^ Significant Differences between Tender Points (*p* < 0.005).

## Data Availability

The data that support the findings of this study are available from the corresponding author (J.A.V-C), upon reasonable request.

## References

[1] Wolfe F., Clauw D.J., Fitzcharles M.-A., Goldenberg D.L., Häuser W., Katz R.L., Mease P.J., Russell A.S., Russell I.J., Walitt B. (2016). 2016 Revisions to the 2010/2011 fibromyalgia diagnostic criteria. Semin. Arthritis Rheum..

[2] Palomo-López P., Becerro-De-Bengoa-Vallejo R., Elena-Losa-Iglesias M., López-López D., Rodríguez-Sanz D., Cáceres-León M., Calvo-Lobo C. (2019). Relationship of Depression Scores and Ranges in Women Who Suffer from Fibromyalgia by Age Distribution: A Case-Control Study. Worldviews Evid. Based Nurs..

[3] Clauw D.J. (2014). Fibromyalgia: A clinical review. JAMA.

[4] Langhorst J., Klose P., Musial F., Irnich D., Häuser W. (2010). Efficacy of acupuncture in fibromyalgia syndrome—a systematic review with a meta-analysis of controlled clinical trials. Rheumatology.

[5] Marques A.P., Santo A.D.S.D.E., Berssaneti A.A., Matsutani L.A., Yuan S.L.K. (2017). Prevalence of fibromyalgia: Literature review update. Rev. Bras. Reum. Engl. Ed..

[6] Fitzcharles M.-A., Ste-Marie P.A., Goldenberg D.L., Pereira J.X., Abbey S., Choinière M., Ko G., Moulin D.E., Panopalis P., Proulx J. (2013). 2012 Canadian Guidelines for the Diagnosis and Management of Fibromyalgia Syndrome: Executive Summary. Pain Res. Manag..

[7] Neumann L., Buskila D. (2003). Epidemiology of fibromyalgia. Curr. Pain Headache Rep..

[8] Sánchez A.M.C., López H.G., Sánchez M.F., Mármol J.M.P., Aguilar-Ferrándiz M.E., Luque-Suarez A., Matarán-Peñarrocha G.-A. (2019). Improvement in clinical outcomes after dry needling versus myofascial release on pain pressure thresholds, quality of life, fatigue, pain intensity, quality of sleep, anxiety, and depression in patients with fibromyalgia syndrome. Disabil. Rehabil..

[9] Montoya P., Pauli P., Batra A., Wiedemann G. (2005). Altered processing of pain-related information in patients with fibromyalgia. Eur. J. Pain.

[10] Desmeules J., Cedraschi C., Rapiti E., Baumgartner E., Finckh A., Cohen P.L., Dayer P., Vischer T.L. (2003). Neurophysiologic evidence for a central sensitization in patients with fibromyalgia. Arthritis Rheum..

[11] Úbeda-D’Ocasar E., Gallego-Sendarrubias G.M., Guodemar-Pérez J., Hervás-Pérez J.P. (2020). Differences between men and women with fibromyalgia. Phys. Med. Rehab. Kuror..

[12] Maurer A.J., Lissounov A., Knezevic N.N., Candido K.D., Knezevic N.N. (2016). Pain and sex hormones: A review of current understanding. Pain Manag..

[13] Harden R.N., Revivo G., Song S., Nampiaparampil D., Golden G., Kirincic M., Houle T.T. (2007). A Critical Analysis of the Tender Points in Fibromyalgia. Pain Med..

[14] Häuser W., Ablin J., Fitzcharles M.-A., Littlejohn G., Luciano J.V., Usui C., Walitt B. (2015). Fibromyalgia. Nat. Rev. Dis. Prim..

[15] Ge H.-Y. (2010). Prevalence of Myofascial Trigger Points in Fibromyalgia: The Overlap of Two Common Problems. Curr. Pain Headache Rep..

[16] Cuschieri S. (2019). The STROBE guidelines. Saudi J. Anaesth..

[17] Monterde S., Salvat I., Montull S., Fernández-Ballart J. (2004). Validacion de la version española del Fibromyalgia Impact Question-naire [Validation of Spanish version of the Fibromyalgia impactquestionnaire]. Rev. Esp. Reumatol..

[18] Jacobs J.W.G., Geenen R., Van Der Heide A., Raske J.J., Bijlsma J.W.J. (1995). Are Tender Point Scores Assessed by Manual Palpation in Fibromyalgia Reliable?:An investigation into the variance of tender point scores. Scand. J. Rheumatol..

[19] Fors E.A., Wensaas K.-A., Eide H., Jaatun E.A., Clauw D.J., Wolfe F., Helvik A.-S. (2020). Fibromyalgia 2016 criteria and assessments: Comprehensive validation in a Norwegian population. Scand. J. Pain.

[20] Karcioglu O., Topacoglu H., Dikme O., Dikme O. (2018). A systematic review of the pain scales in adults: Which to use?. Am. J. Emerg. Med..

[21] Ge H.-Y., Wang Y., Danneskiold-Samsøe B., Graven-Nielsen T., Arendt-Nielsen L. (2010). The Predetermined Sites of Examination for Tender Points in Fibromyalgia Syndrome Are Frequently Associated with Myofascial Trigger Points. J. Pain.

[22] Fillingim R.B., Loeser J.D., Baron R., Edwards R.R. (2016). Assessment of Chronic Pain: Domains, Methods, and Mechanisms. J. Pain.

[23] Tastekin N., Uzunca K., Sut N., Birtane M., Mercimek O.B. (2010). Discriminative value of tender points in fibromyalgia syndrome. Pain Med..

[24] Linton S.J., Shaw W.S. (2011). Impact of Psychological Factors in the Experience of Pain. Phys. Ther..

[25] Fillingim R.B. (2017). Individual differences in pain: Understanding the mosaic that makes pain personal. Pain.

[26] Fillingim R.B., King C.D., Ribeiro-Dasilva M.C., Rahim-Williams B., Riley J.L. (2009). III. Sex, Gender, and Pain: A Review of Recent Clinical and Experimental Findings. J. Pain.

[27] Fillingim R.B.Y., Turk R.P.D.C., Sierra F.K. (2016). Pain in the elderly. Advances in Geroscience.

